# Developing a survey of barriers and facilitators to recruitment in randomized controlled trials

**DOI:** 10.1186/1745-6215-13-218

**Published:** 2012-11-21

**Authors:** Geetinder Kaur, Rosalind L Smyth, Paula Williamson

**Affiliations:** 1 Department of Women’s and Children’s Health, Institute of Translational Medicine, University of Liverpool, Institute of Child Health, Alder Hey Children’s Hospital, Eaton Road, Liverpool, L12 2AP, UK; 2 Department of Biostatistics, Faculty of Health and Life Sciences, University of Liverpool, Brownlow Street, Liverpool, L69 3GS, UK; 3 UCL Institute of Child Health, 30 Guilford Street, London, WC1N 1EH, UK

**Keywords:** Recruitment, Survey, Questionnaire, Clinical trials

## Abstract

**Background:**

Recruitment to randomized controlled trials is known to be challenging. It is important to understand and identify predictors of good or poor accrual to a clinical trial so that appropriate strategies can be put in place to overcome these problems and facilitate successful trial completion. We have developed a survey tool to establish the recruitment experience of clinical teams regarding facilitators and barriers to recruitment in a clinical trial and describe herein the method of developing the questionnaire.

**Methods:**

A literature search was conducted to identify studies that have explored facilitators and barriers to recruitment, and a list of potential factors affecting recruitment to a clinical trial was generated. These factors were categorized in terms relating to the (i) trial, (ii) site, (iii) patient, (iv) clinical team, (v) information and consent and (vi) study team. A list was provided for responders to grade these factors as weak, intermediate or strong facilitators or barriers to recruitment.

**Results:**

A web-based survey questionnaire was developed. This survey was designed to establish the recruitment experience of clinical teams with regard to the perceived facilitators and barriers to recruitment, to identify strategies applied to overcome these problems, and to obtain suggestions for change in the organization of future trials. The survey tool can be used to assess the recruitment experience of clinical teams in a single/multicenter trial in any clinical setting or speciality involving adults or children either in an ongoing trial or at trial completion. The questionnaire is short, easy to administer and to complete, with an estimated completion time of 11 minutes.

**Conclusions:**

We have presented a robust methodology for developing this survey tool that provides an evidence-based list of potential factors that can affect recruitment to a clinical trial. We recommend that all clinical trialists should consider using this tool with appropriate trial-specific adaptations to monitor and improve recruitment performance in an ongoing trial or conduct the survey at trial completion to gather information on facilitators and barriers to recruitment that can form the basis of interventions and strategies to improve recruitment to future clinical trials.

## Background

Recruitment to randomized controlled trials is known to be challenging. However, effective and timely recruitment of appropriate and adequate numbers of research participants is essential for the successful completion of a trial and generation of valid results.

Prolonged or inefficient recruitment can have adverse scientific, economic and ethical consequences [1]. Failure to achieve the target sample size can lead to a reduction in the statistical power of a study. An underpowered study may report clinically important effects to be statistically non-significant and result in delay or non-implementation of a clinically effective intervention and delay in identification of non-effective interventions. Prolonged recruitment results in increased time or cost extensions and may result in premature termination of trials. Studies that terminate prematurely or fail to reach adequate statistical power raise ‘ethical’ concerns as trialists have exposed the participants to an intervention with uncertain benefit and may still be unable to determine whether the intervention does more harm than good at trial completion [2].

As under-recruitment is a common cause for trial failure, it is important to understand and identify the predictors of good or poor accrual to a clinical trial so that appropriate strategies can be put in place to overcome these problems and facilitate successful trial completion.

Several studies have examined recruitment experience from a number of perspectives. There are reports by trialists describing their recruitment experience, methods and strategies [3-10]. There are reports on recruitment and participation of under-represented populations such as minorities [11,12], adolescents, and young adults in cancer trials [13]. Studies have tried to assess parents’ or families’ reasons for participation or non-participation in trials [14-16], and there are several reports of surveys and interviews with parents or patients investigating the same [17-24].

Surveys and interviews with clinical teams have investigated reasons for considering patients unsuitable for a trial [25], reasons for not entering eligible patients [26], and difficulties with recruitment to the trial [27,28]. Caldwell *et al*. [29] conducted focus group discussions with sixteen pediatricians and five trainees from a pediatric teaching hospital to evaluate pediatricians’ attitudes toward participation of children in randomized controlled trials and to identify potential barriers to participation. A number of studies have explored barriers to trial participation from patients’ and clinicians’ perspectives. Systematic reviews of studies [30-32] reporting barriers to participation in cancer trials have identified various patient and clinician-related barriers. Fayter *et al*. [33] conducted a systematic review to investigate the barriers, modifiers and benefits of participation in randomized controlled trials of cancer therapies as perceived by health care providers or patients and identified system-related or organizational barriers, trial design-related and health care provider barriers. Twenty-five studies explored barriers to participation from the health care perspective, with eight studies investigating recruitment to specific trials and seventeen studies investigating attitudes to trials in general. However, the authors concluded that the studies were of poor methodological quality and identified threats to internal validity in terms of potential for selection bias, non-justification of sample size, lack of reliability and validity of research instrument, and problems of data collection. None of the included surveys in this systematic review provided a comprehensive list of facilitators and barriers to recruitment.

Cook *et al*. [34] conducted a survey to explore the experiences, beliefs and practices of Critical Care Trials Groups regarding the effectiveness, feasibility and ethics of strategies to enhance enrollment and views on co-enrollment of critically ill children and adults into one or more clinical studies. Fernandez *et al*. [35] conducted a trial-specific survey to explore the physicians’ and parents’ barriers to enrollment in the Children’s Oncology Group’s study of very low risk Wilm’s tumor. Spaar *et al*. [36] conducted a postal survey among recruiting physicians in a multicenter trial of respiratory rehabilitation in patients with chronic obstructive pulmonary disease to identify and weigh barriers to recruitment to the trial. The survey questionnaire comprised barriers identified in literature that were applicable to the trial and concerns raised by recruiting physicians during the recruitment process. Studies [25-28,35] examine barriers to recruitment in the context of a specific trial or a specific population, and the survey questionnaires have been developed as trial or speciality specific [34]. Spaar *et al*. investigated some general barriers to recruitment as well, but not comprehensively, and recruitment facilitators were not identified. We have developed a survey instrument that can be used to investigate the experience of clinical teams with regard to both facilitators and barriers to recruitment to a single/multicenter clinical trial in any clinical setting or speciality. The survey questionnaire is evidence-based and has the potential to explore the generic factors affecting recruitment to a clinical trial with the scope of adding trial- and speciality-specific questions, thus providing a reliable tool and systematic approach to recognition and management of recruitment problems. To the best of our knowledge, there is no such existing recruitment survey tool, and we describe herein the method of developing this survey questionnaire.

## Methods

### Survey design

**Figure 1:**
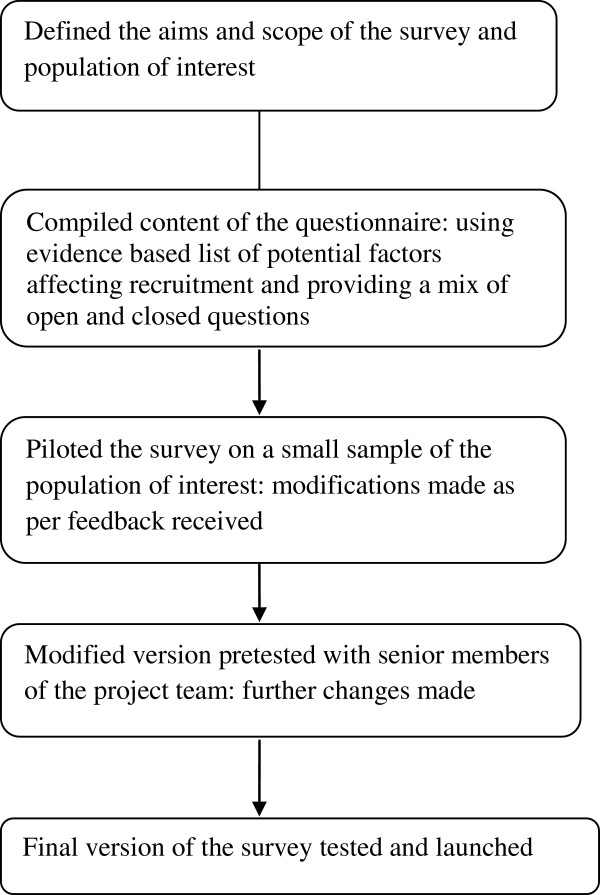
Process of developing the questionnaire.

The survey has been designed as an online questionnaire to be completed by study teams involved with recruitment to a trial. The process of developing the questionnaire is presented in Figure 1. The survey is divided into four main sections to collect information about the site and study role of the responders, the perceived facilitators and barriers to recruitment, strategies applied to overcome the problems and suggestions for changes in organization of future trials.

Free-text space is provided for participants to enter information on their site or center of recruitment, their role in the study, whether they opened to recruitment, and their duration and period of involvement if they were not involved for the whole trial recruitment period. It is possible to add questions for collecting further information on the estimated number of eligible patients, the numbers expected to be randomized, and the hospital policy on recruitment to the study. Skip logic can be applied to direct questions selectively to responders depending on their response to previous questions so that they may skip questions that do not apply to them. The second section provides the survey participants with preformed lists of potential factors affecting recruitment that could act as facilitators or barriers to recruitment.

### Writing the questionnaire

A list of potential facilitators and barriers affecting recruitment to randomized controlled trials was made from a review of existing literature on the subject. A literature search on Medline using the search terms ‘recruitment’, ‘enrolment’ combined with the AND connector to search terms for ‘clinical trials’ and ‘randomised controlled trials’ identified the major reviews on the subject that were used to develop the list of factors.

The reviews used to design the survey questionnaire are briefly described below. The process of selecting and classifying the factors for inclusion in the survey questionnaire is presented in Table 1. The Health Technology Assessment (HTA) report, ‘Factors that limit the quality, number and progress of randomized controlled trials’ by Prescott *et al*. [37] reported patient and clinician barriers to participation in randomized controlled trials. The authors of that report conducted a systematic review of studies that reported problems related to recruitment of clinicians and patients to clinical trials and identified the important barriers.

**Table 1:** Deriving the factors affecting recruitment from facilitators and from barriers described in literature

**Prescott *et al. *[**[37]**]**		
*Barriers to participation in clinical trials: patient and clinician barriers*		
Barriers	Classification	Factor derived
*Patient barriers*		
*Additional demands of the randomized controlled trials (RCT) on the patient*	Patient level factors	Additional trial investigations
Additional procedures, additional appointments, time pressures, venepuncture, inpatient hospital stays, discomfort from medical procedures, length of study, worry about experimentation, uncomfortable procedures, travel and travel costs, extra costs		Additional travel and extra costs
		Duration of trial and follow- up
*Patient preference for a particular treatment*	Patient level factors	Patients’/parents’ preference for a particular treatment
Patients not wanting to change medication, not to take placebo, not to take experimental medication, not to take any medication, patient request for a specific intervention, strong patient preference for one treatment option		Patients’/parents’ attitudes towards taking experimental medicine or placebo
*Aversion to treatment choice by random allocation*		Treatment choice by random allocation
*Worry about uncertainty*	Patient level factors	Patients’/parents’ concerns about side effects of new drug
Efficacy of treatment on offer is unproven, distrust of hospital or medicine, fear of unknown		
*Concerns about information and consent*	Information and consent related factors	Amount and complexity of trial information provided
Amount of information provided to research participants, wording of information, complexity of information provided, different forms of information presentation: written /verbal/video, limited reading skills and English not being the primary language, clinicians’ experience, difficulty in giving information, worry about level of information required and that information may be frightening, consent procedure barrier to recruitment	Patient level factors	Clarity in presentation of trial information
	Clinical team factors	Experience and training of clinical team seeking consent
		Social and emotional dynamics of trial discussion
		Consent rate
		Language or cultural barrier
		Difficulty in approaching patents for consent
*Clinician barriers*		
*Time constraints*	Clinical team factors	Clinical workload
Time pressures from usual clinical practice, time demands of recruitment and follow-up		
*Staffing and training*	Clinical team factors	Research experience of clinical team
Lack of trained staff, no additional support, lack of research experience in clinicians, lack of available support staff		Availability of designated research team
		Availability of research staff out of hours
		Presence of designated research nurse/practitioner
*Rewards and recognition*	Excluded	Information available from the Chief Investigator
Economic incentives		
*Impact on doctor patient relationship* fear of Adverse effect on doctor-patient relationship, perceived conflict in their role as clinicians and researchers	Clinical team factors	Clinician attitude to involving patients in research
*Concern for patients*		
Concern about treatment toxicity, side effects, burden of trial for patients including travel distance and costs, reluctance to recruit severely ill patients		
*Problems in complying with the protocol*	Clinical team factor	Clinician preference for a particular treatment
	Trial level factor	Study protocol compared to clinical practice
**Campbell *et al. *[**[38]**]**		
*Hypothesis of factors tested for association with recruitment success*		
Trials with complex trial design do not recruit as well as simple trials	Trial level factor	Trial design
Less well-funded trials do not recruit well	Trial level factor	Funding
Trials without dedicated trial management expertise do not recruit as well as those with trial management expertise	Trial level factor	Trial management
Trial with multidisciplinary input recruit better than those that do not have this input	Excluded	Information available from the Chief Investigator
Trials with consumer involvement recruit better than those that do not	Excluded	Information available from the Chief Investigator
Trials that have a successful pilot phase recruit better than those that do not have a pilot phase	Trial level factor	Previous feasibility assessment
		Previous pilot trial
Trials that have dedicated paid local coordinators recruit better than those that do not		Trial management
Cancer trials recruit better than non cancer trials	Trial level factor	Being a drug/cancer trial
Drug trials recruit better than non-drug trials		Being a drug/cancer trial
Trials funded through a response-mode funding have different recruitment rates to those funded through a commissioned process	Trial level factor	Funding
*Reasons for delays in recruitment to the included cohort of trials*		
Problems with central staff, local research staff, internal problems (for example, staff)	Site level factor	Number of trained staff
	Clinical team factor	Motivation of clinical team
Local clinical arrangements, merging/reorganization of trusts, major relocation of services, department policies	Site level factor	Local clinical arrangements
Funding issues	Trial level factor	Funding
Delays in ethical clearance	Excluded	Information available from the Chief Investigator
Research and Development (R&D) delays, time delay since grant application		
Delays in supply of drug/placebo	Excluded	Information available from the Chief Investigator
Adverse publicity about medical research, external problem (for example, publicity)	Trial level factor	Publicity by the trial team
		External publicity
Setting up general practitioner (GP) practices took longer than anticipated	Site level factor	Time to open up site
Simultaneous other local research projects, competing research, conflict with other trials	Site level factor	Competing local research projects
Delays due to changes in data legislation, changes in technology	Excluded	Information available from the Chief Investigator
Fewer eligible than expected, smaller percentage agreeing to participate, recruitment targets too ambitious	Trial level factor	Lack of pilot/feasibility assessment
	Site level factor	Recruitment target
Absence of perceived clinical equipoise	Trial level factor	Clinical equipoise
Issues with procedures/interventions, trial process too demanding	Patient level factor	Additional trial investigations
Complexity of trial design, trial methodology considered too complex	Trial level factor	Trial design
Conflicting workload pressures, long waiting lists, additional theatre time required	Clinical team factor	Clinical workload
Language/written English difficulties	Patient level factor	Language or cultural barrier
Treatment preferences	Patient level factor	Patients’/parents’ preference for a particular treatment
	Clinical team factor	Clinician preference for particular treatment
Research not considered as priority	Clinical team factor	Perceived importance of research generally in clinical practice
		Perceived importance of the particular research question
No local access to intervention	Patient level factor	Intervention available only in the trial
*Case studies of trials: common factors in the successes of part B trials*		
*Facilitator*	*Classification*	*Factor derived*
Important/interesting research question, topic important, urgent need for research, important question, timely and managed to roll several questions into one study	Clinical team factor	Perceived importance of the particular research question
Good design/good protocol, pragmatic study	Trial level factor	Trial design
		Study protocol compared to clinical practice
Clinicians keen to recruit to trial	Clinical team factor	Motivation of clinical team
		Clinician attitude to involving patients in research
Drugs already tested, so easy to explain to patients	Patient level factor	Familiarity with experimental treatment
Did not demand extra effort from patients, Impact on practice running and costs minimized, minimizing work for health professionals	Patient level factor	Additional trial demands
No competing trials for those centers/patients	Site level factor	Competing local research projects
Drugs not available outside the trial	Patient level factor	Intervention available only in the trial
Excellent trial management, trial units helpful, caring, annual meetings for all concerned, role of trial steering group	Trial level factor	Trial management
Good planning and organization by Clinical Trials Support Unit (CTSU), CTSU responsive, efficient, central organization of many aspects of research		
Good communication between trial team and clinicians, flexibility of trial teams	Study team factor	Communication and coordination among study team members at site
Good public relations/feedback/updates	Trial level factor	Trial publicity
Good funding, National Health Service (NHS) funding	Trial level factor	Funding
Trial run by good team/infrastructure, Principal Investigator (PI) well respected, PIs worked hard to keep collaborators on board, trial team communicative, responsive and alert to problems. Communication within team, between team and collaborating clinicians	Study team factors	Motivation of the study team at site
	Clinical team factor	Research experience of PI and study team members at site
Good trial team, good research assistants		Communication and coordination among study team members at site
Team worked hard at how to explain the study to patients		Communication and coordination between study team at site and Clinical Trials Unit (CTU)
		Research experience of clinical team
		Communication skills of clinical team
Role of research nurse	Clinical team factor	Presence of designated research nurse/practitioner
Study included everybody	Trial level factor	Patient inclusion criteria
**Toerien *et al*. [**[39]**]**		
Study design, number of arms, control: active/placebo	Trial level factor	Trial design
Single/multicenter	Excluded	Information will be present
Intervention: drug/surgery/allied/others	Trial level factor	Being a drug/cancer/surgical/-----trial
Funding source	Trial level factor	Funding
**Caldwell *et al*.[**[40]**]**		
*Recruitment strategies*		
Novel trial designs	Trial level factor	Trial design
Recruiter differences	Information and consent related factors	Experience and training of doctors clinical team seeking consent
		Senior doctors and nurses seeking consent
Financial incentives for patients/participants	Excluded	Monetary incentives not acceptable for clinical research in United Kingdom
Methods of providing information	Information and consent related factors	Amount and complexity of information provided
	Patient level factor	Clarity in presentation of trial information
		Consent rate
**Treweek *et al*.[**[2]**]**		
*Recruitment strategies*		
Design changes	Trial level factor	Trial design
Modification to the consent form or process	Patient level factor	Consent rate
Modification to the approach made to potential participants	Information and consent related factors	Amount and complexity of information provided
		Clarity in presentation of trial information
		Senior doctors and nurses seeking consent
Financial incentives for patients/participants	Excluded	Monetary incentives not acceptable for clinical research in United Kingdom
Modification to the training given to recruiters	Information and consent related factors	Experience and training of clinical team seeking consent
Greater contact between trial coordinator and trial sites	Trial level factor	Trial management

The HTA report ‘Recruitment to randomised trials: strategies for trial enrolment and participation study: The STEPS study’ aimed to identify the factors associated with good and poor recruitment to multicenter trials (38). They conducted an epidemiological review (The STEPS study Part A) of a cohort of trials funded by the Medical Research Council (MRC) and the National Health Service Health Technology Assessment (NHS HTA) program between January 1994 and December 2002. They tested hypotheses of factors for association with recruitment success in the cohort of multicenter randomized controlled trials included in the review, described patterns of recruitment and reported trialists’ perceptions of factors associated with good or poor recruitment. The study also reported the reasons for delay in recruitment and early and late participant recruitment problems in the included cohort of trials based on the trialists’ reports submitted to the funding bodies. The STEPS study (Part B) reported case studies of trials that recruited successfully and had particularly interesting lessons for recruitment. This aim of this part of the study was to gain role specific and location specific insights to the four included trials by interviewing 45 individuals in total across the four trials with different internal perspectives. Four key stages of a trial that may affect recruitment were identified: 1) foundation work involving engagement of collaborators, establishing scientific rigor, funding and financial considerations, 2) recruitment processes, 3) delivery of care and 4) delivery of research. Common factors in the success of the included trials were reported based on analysis of themes identified in the four key stages and from the responses of the interviewees. Toerien *et al*. [39] reviewed the recruitment and retention rates in randomized controlled trials published in six major journals between July and December 2004 and investigated the association of these rates with trial characteristics such as study size, number of arms, single/multicenter, treatment focus (drug/surgery/allied/others), active/placebo control, time to assessment and type of funding. The Cochrane systematic review on strategies to improve recruitment to randomized controlled trials [2] identified 45 randomized and quasi-randomized controlled trials of interventions directed at potential participants or clinicians, which aimed to improve recruitment of participants to clinical trials. These interventions were divided into six categories: design change, modification to the consent form or process, modification to the approach made to potential participants, financial incentives for participants, modification of training given to recruiters and greater contact between trial coordinator and trial sites. The systematic review of strategies for increasing recruitment to randomized controlled trials by Caldwell *et al*. [40] looked at the effect of recruitment interventions such as novel trial designs, recruiter differences, incentives and different methods of providing trial information on recruitment success in randomized clinical trials.

From the facilitators and barriers reported in the above studies (37,38) and the potential factors and interventions tested for association with recruitment success (2,38-40), a list of potential factors affecting recruitment was generated by classifying the facilitators and barriers into various categories. This process is presented in Table 1. The factors that were generic and expected to operate commonly at all sites were classified as ‘trial level factors’. These included factors such as funding for the trial, trial design, choice of patient inclusion criteria, type of intervention, previous pilot/feasibility assessment, perception of clinical equipoise, publicity about the trial, trial management, *etcetera* The factors which could operate differentially between sites were classified as ‘site level factors’ and included factors such as time to open up site, recruitment target, local clinical arrangements, number and availability of trained staff, competing research projects and local research culture to list a few. We excluded factors for which objective information is available such as delays in ethical clearance, research and development delays, and problems with supply of investigational drug/placebo *etcetera* The various facilitators and barriers relating to patients’ and clinicians’ participation in clinical trials, as described in the above studies were listed under ‘patient related and clinical team related factors’. The factors related to providing information to patients and seeking consent such as amount and complexity of trial information, clarity in presentation of trial information, time and setting of consent seeking and role and seniority of person seeking consent were categorized separately as ‘information and consent related factors’. Lastly, the study team factors such as motivation and research experience of study team, communication and coordination between research teams were presented. Each category formed a separate question in the survey questionnaire to help the participants think through the issues arising during recruitment to the trial.

This section of the survey could be designed to elicit only barriers, only facilitators, or both barriers and facilitators to recruitment. In order to decrease the length of the survey and capture information on both facilitators and barriers in a common question, the factors were reworded such that they could apply both as a facilitator or barrier depending on whether they boosted or hindered recruitment respectively. The questions in this section were designed to obtain graded responses from ‘-3’ to ‘+3’ depending on whether the factor was perceived to be a strong (−3), intermediate (−2) or weak barrier (−1), 0 if thought to be not applicable and weak (+1), intermediate (+2), or strong facilitator (+3).

Open-type questions were provided to obtain information on the various strategies applied to overcome the problems and for participants to express their reflective experiences and views on how trials could be organized differently in the future to improve recruitment.

### Pretesting/piloting the questionnaire

The paper version of the questionnaire was sent for piloting to a small sample of five people. Three out of the five people (60%) responded. The initial version had separate lists of facilitators and barriers and participants were asked to identify the top five in each list. Two out of the three respondents found the questionnaire lengthy, difficult to complete and it took them 35 to 40 minutes to do so. Some questions were thought to be ambiguous and there was a suggestion for use of computers to enhance the presentation and make it easier to complete.

After the pilot, the questionnaire was modified. The length of the questionnaire was reduced by combining the facilitators and barriers into a single list of factors that could be graded as either in the same question. Efforts were made to provide an evidence-based list of factors affecting recruitment while taking measures to keep the length of the survey and time of completion within reasonable limits. Factors for which objective information was believed to be available from other sources, such as delays in ethical or research and development approval or problems with supply of investigational drug/placebo were excluded from the questionnaire. However, free text space for additional comments was provided at the end for responders to note any issues not covered in the survey.

The factors were reworded so that they were simpler and clearer. An online version was created by using survey software (http://www.surveygizmo.com). The questions were arranged such that they had a logical flow. Each category of factors was arranged as small separate sections on a webpage for better presentation and ease of completion. The participants could easily navigate forward and backward to revisit a section if they needed to and the completion time was reduced to 10 to 15 minutes. The survey instrument (Additional file 1: Figure S1) has been used to investigate the recruitment experience of clinical teams in a large multicenter randomized controlled trial with children in the United Kingdom (MAGNETIC trial). The response rates are presented here, but the results of the survey for identified facilitators and barriers, the strategies applied to overcome these barriers and the suggestions for better organization of trials to improve recruitment, will be published separately. The MAGNETIC trial was a large multicenter, randomized, double-blind placebo-controlled trial evaluating the role of nebulized magnesium in acute severe asthma in children. It recruited over 500 children over a period of 26 months from 30 sites across the United Kingdom. The recruitment experience varied across sites and the opportunity to gather information about this study was believed to be important. There were three other sites that had opened to recruitment but did not recruit any patients and four others where efforts were made to set up the trial but did not open to recruitment. The survey was sent to all 37 sites as recruitment problems and perspectives of clinical teams were envisaged to be different at different sites. The names and contact email addresses of the potential responders were obtained from the delegation logs. This included all clinical team members including the Principal Investigators (PIs), research nurses (RNs),medical practitioners, nursing staff and nurse practitioners, who were delegated to be involved with recruitment to the trial. The survey was emailed to 522 contacts at all 37 sites. Since contact email addresses were expected to change over the course of the trial, especially for the various clinical staff, we selectively pursued responses from the Principal Investigator and at least one research nurse at each site.

## Results and discussion

### Results

The first section was designed to collect information about the responder’s study role, site of recruitment and period and duration of his or her involvement in the trial. Personal information such as name was not collected; instead, each survey participant was assigned a unique identification code for purposes of data collection and analysis. Information on sites and study role was also used to examine representation in responses.

The second section provided a comprehensive list of factors affecting recruitment and the survey participants were asked to rate each factor from ‘-3’ to ‘+3’ as described. Each factor could be assigned only one score. This question was designed in this format to enable us to deduce the most commonly identified strong barriers and facilitators and also to enable us to calculate average scores for each factor. The last section had open-ended questions to gain information on the interventions applied and to collate reflective experiences and suggestions of the study team to improve recruitment with space for additional comments.

The project team sent initial invitations that described the aims and objectives of the survey, provided information about the survey, and requested participation in the survey to the potential participants. The email provided the link to the online questionnaire, stated that participation in the survey was voluntary, and that consent would be assumed by the person completing the questionnaire. The responders were reassured that no personal information such as names would be collected, no site will be identified in any publication and confidentiality of data will be maintained.

A total of 169 complete responses were obtained. We achieved a PI and one research nurse response per site from 86% and 94% of sites respectively. The response rates for PIs and RNs (one per site) are summarized in Table 2.

**Table 2:** Response rates for survey of facilitators and barriers to recruitment to the MAGNETIC trial

**Sites (n = 37)**	**Principal investigators n (%)**	**Research nurses n (%)**
Sites that recruited (n = 30)	28 (93%)	28 (93%)
Sites that opened but did not recruit (n = 3)	2 (67%)	1 (100%)^a^
Sites that never opened (n = 4)	2 (50%)	1 (100%)^a^
Overall response	32 (86%)	30 (94%)

^a^Research nurse present at only one site.

One hundred percent of the responders completed all questions ranking the factors affecting recruitment as facilitators or barriers. Of those responders 108 of 169 (64%) responded to the open question on interventions or strategies applied to overcome the barriers identified. Free-text suggestions were obtained from 115 of the 169 (68%) respondents on future reorganization of the trial to improve recruitment.

## Discussion

A survey is a systematic method of collecting data from a population of interest, usually through the use of a structured and standardized questionnaire [41]. The methods of conducting survey research can be interviews, either face to face or via telephone, or using postal or electronic questionnaires. The advantages of a participant-completed questionnaire over an interview are that it is quicker and cheaper, avoids interviewer bias and allows respondents to record their responses privately even to sensitive issues. The disadvantages are that questions may be misunderstood or not fully answered by the respondent. There is a greater need to use closed questions to ensure consistency in the range of answers and for ease of analysis [42]. E-surveys offer a number of advantages over paper or telephone survey techniques in terms of less time and cost requirements, better accuracy in terms of fewer data transcription errors, faster creation and delivery, enhanced presentation and a higher response rates. The potential disadvantages include response bias resulting from unequal access to internet, issues of authenticity, data security and confidentiality, and respondent non-response or procrastination [43].

For these reasons, the recruitment survey questionnaire was developed as an online tool. Care was taken to avoid errors due to respondent misinterpretation of questions by phrasing the questions in a simple and clear manner. A mix of open and closed questions was provided to obtain accurate responses but also to provide respondents the freedom to express their views. Efforts were made to make the survey user-friendly by arranging the questions in a logical order and reducing the length of the survey. However, as for any other survey instrument the generation of useful results depends on a good response rate from a representative sample of the population of interest and obtaining true and accurate responses from participants. For the MAGNETIC trial, the recruitment survey questionnaire achieved a response from the Principal Investigator at 86% of the sites and at least one research nurse response from 94% of the sites. All the respondents completed the ranking of factors as facilitators or barriers, and free-text responses on interventions to overcome barriers and suggestions for future re-organization were obtained from 64% and 68% of the responders, respectively.

Recruitment to a clinical trial and its conduct is shaped by various internal and external forces including the shifting dynamics at sites because of changes in jobs and roles of staff including periodic turnover of trainee doctors every few months and change in policies at the hospital or trust level. Understanding the working of individual trials and of trial teams at various sites in a multicenter trial, with their unique challenges, as well as the responses of the research teams to these challenges, can provide important information that can be used to inform the design and conduct of future trials [38].

This survey questionnaire could be a very useful tool to investigate the recruitment experience of clinical teams and to identify facilitators and barriers to recruitment to a single or multicenter clinical trial in any clinical setting or speciality involving adults or children. It provides a common list of questions to participants at multiple sites and can be used to elicit the facilitators and barriers to trial participation in general, and can also be adapted and modified by adding trial specific questions and highlighting trial- and speciality-specific recruitment issues. It provides a mix of open and closed questions and allows for free-text space for participants to share their experiences and make reflective comments. It is designed to gather data from people with a range of responsibilities related to recruitment to the trial. It can be aimed at staff directly involved with recruitment but can also be extended to other staff that facilitate recruitment or are involved indirectly to gain an insight into their perspective on issues around recruitment to the trial. The survey can be easily sent to a large number of participants at the same time. It can be used to gauge role- and site-specific perceptions of the research team and can provide a detailed understanding of the various factors affecting recruitment in addition to providing information from other monitoring tools such as screening logs.

This survey tool was designed to be used at the end of the recruitment phase of a study to identify useful lessons for future research and to other trialists. It could be used however, with some modification, in the pre-trial phase to identify potential problems or in the early and middle recruitment phases when observed participation rate is lower than expected. During a trial, the study team will often contact under-recruiting sites to obtain information about problems encountered or will contact higher than average recruiting sites to identify facilitators. This tool would provide a more systematic approach to the collection and consideration of such information, ensuring that all evidence-based barriers and facilitators are reviewed by the site in their response, so that appropriate strategies can be implemented to overcome the problems identified. If the survey is to be undertaken during the recruitment phase to identify modifiable aspects of the process, factors such as the time taken to open the site and whether there was a previous feasibility or pilot study would not be relevant.

Since recruitment performance is usually variable at different sites, it can be used to investigate the various site-specific issues. This will not only provide a detailed understanding of the internal milieu of the trial but also provides the opportunity for comparison of responses between successful and non-successful sites. Identification of facilitators or barriers and strategies applied at sites with successful recruitment in comparison to less successful sites may highlight some modifiable differences, which can form the basis of interventions and strategies to boost recruitment to an ongoing clinical trial or provide useful lessons for designing and conducting future trials. The survey questionnaire has some potential limitations. Being a subjective tool, it is prone to responder misinterpretation, and the authors encourage trialists to pilot the questionnaire with a sample of their trial team prior to use to ensure consistent understanding of the listed factors. Though it has been designed to provide an evidence-based list of generic factors that affect recruitment to clinical trials, the authors would again encourage trialists to think about other anticipated or observed trial-specific issues and modify and adapt the questionnaire before use, taking into consideration the type of trial and the stage of recruitment. The length of the survey can be reduced further by excluding factors that are thought to be not relevant to a particular trial.

## Conclusions

Recruitment to clinical trials is a common problem. Understanding the working of individual trials and identification of recruitment facilitators and barriers can provide important information to improve recruitment to the trial and to help with better design in future trials. We have developed a recruitment survey tool providing an evidence-based list of generic factors that can affect recruitment to a clinical trial. It can be used in any clinical setting or speciality involving adults or children and can be modified by adding trial-specific issues. Clinical trialists may identify other trial specific recruitment issues and make appropriate modifications. Piloting the survey questionnaire with the trial teams prior to use is advisable to ensure consistency in the understanding of terminology.

This survey tool is a potentially useful tool that can help trialists to approach recruitment problems in a systematic manner. It has been used successfully to elicit barriers and facilitators to recruitment in the MAGNETIC trial but would need further evaluation in other trials. If proved to be useful, we would recommend that clinical trialists consider using and adapting this tool to monitor and improve recruitment performance in an ongoing trial or to conduct the survey at trial completion to gather information on facilitators and barriers to recruitment. This information will form the basis of interventions and strategies to enhance recruitment to clinical trials and channelize resources effectively to counter the problems of under-recruitment in future clinical research.

## Abbreviations

HTA: Health Technology Assessment; MRC: Medical Research Council; NHS: National Health Service.

## Competing interests

The authors declare that they have no competing interests.

## Authors’ contributions

GK reviewed the literature, designed and developed the survey tool, and drafted the manuscript. RS contributed to survey design, participated in critical revision of the manuscript, and gave final approval of version to be published. PW conceived the initial idea, contributed to survey design, participated in critical revision of the manuscript and gave final approval of version to be published. All authors read and approved final manuscript.

## Supplementary Material

Additional file 1 **Figure S1.** Recruitment survey. Click here for file
