# Supplementary material for: Developing a survey of barriers and facilitators to recruitment in randomized controlled trials
Source: Trials. 2012 Nov 21;13:218. doi: 10.1186/1745-6215-13-218 (PMC3563446; doi:10.1186/1745-6215-13-218)
Supplement: Additional file 1 — Figure S1. Recruitment survey. [file 1745-6215-13-218-S1.docx]

**Figure S1: Recruitment survey**

### 1. Please enter your ID number

____________________________________________

#### 2. Please indicate your role with regards to recruitment to the trial

[ ] Site lead/PI

[ ] Medical practitioner

[ ] Research nurse

[ ] Other

### Please describe your role in relation to recruitment to the trial

____________________________________________

### 3. Which site/hospital were you recruiting from?

____________________________________________

#### 4. Have you been involved for the whole trial period?

[ ] Yes

[ ] No

#### Was this during

[ ] setup/early recruitment period

[ ] once trial established at site

### How long were you involved in recruiting for the trial? (approximately, in months)

#### 5. Trial level factors affecting recruitment

#### Listed below are *trial level factors* that commonly affect recruitment. Please indicate whether a listed factor was a facilitator or barrier to recruitment to the trial and rate them from -3 to +3 as below: -3 strong barrier -2 intermediate barrier -1 weak barrier 0 not applicable +1 weak facilitator +2 intermediate facilitator +3 strong facilitator

| Factors | -3 | -2 | -1 | 0 | +1 | +2 | +3 |
| --- | --- | --- | --- | --- | --- | --- | --- |
| Funding |  |  |  |  |  |  |  |
| Trial design |  |  |  |  |  |  |  |
| Patient inclusion criteria |  |  |  |  |  |  |  |
| Being a drug trial |  |  |  |  |  |  |  |
| Study protocol compared to clinical practice |  |  |  |  |  |  |  |
| Clinical equipoise |  |  |  |  |  |  |  |
| Previous feasibility assessment |  |  |  |  |  |  |  |
| Previous pilot trial |  |  |  |  |  |  |  |
| Publicity by the trial team |  |  |  |  |  |  |  |
| External publicity |  |  |  |  |  |  |  |
| Trial management |  |  |  |  |  |  |  |
| Protocol amendments |  |  |  |  |  |  |  |
| Seasonal variation |  |  |  |  |  |  |  |

|  |  |  |  |  |  |  |
| --- | --- | --- | --- | --- | --- | --- |
|  |  |  |  |  |  |  |
|  |  |  |  |  |  |  |
|  |  |  |  |  |  |  |
|  |  |  |  |  |  |  |
|  |  |  |  |  |  |  |
|  |  |  |  |  |  |  |

**6. Site level factors affecting recruitment**

#### Listed below are *site level factors* that commonly affect recruitment. Please indicate whether a listed factor was a facilitator or barrier to recruitment to the trial and rate them from -3 to +3 as below: -3 strong barrier -2 intermediate barrier -1 weak barrier 0 not applicable +1 weak facilitator +2 intermediate facilitator +3 strong facilitator

| Factors | -3 | -2 | -1 | 0 | +1 | +2 | +3 |
| --- | --- | --- | --- | --- | --- | --- | --- |
| Time to open up site |  |  |  |  |  |  |  |
| Recruitment target |  |  |  |  |  |  |  |
| Time to complete administrative work related to the trial |  |  |  |  |  |  |  |
| Number of trained staff |  |  |  |  |  |  |  |
| Local clinical arrangements |  |  |  |  |  |  |  |
| Choice of recruitment setting |  |  |  |  |  |  |  |
| GCP training |  |  |  |  |  |  |  |
| Data collection process |  |  |  |  |  |  |  |
| Competing local research projects |  |  |  |  |  |  |  |
| Local research culture |  |  |  |  |  |  |  |

|  |  |  |  |  |  |  |
| --- | --- | --- | --- | --- | --- | --- |
|  |  |  |  |  |  |  |
|  |  |  |  |  |  |  |
|  |  |  |  |  |  |  |
|  |  |  |  |  |  |  |
|  |  |  |  |  |  |  |
|  |  |  |  |  |  |  |
|  |  |  |  |  |  |  |
|  |  |  |  |  |  |  |
|  |  |  |  |  |  |  |

**7. Patient level factors affecting recruitment**

#### Listed below are *patient level factors* that commonly affect recruitment. Please indicate whether a listed factor was a facilitator or barrier to recruitment to the trial and rate them from -3 to +3 as below: -3 strong barrier -2 intermediate barrier -1 weak barrier 0 not applicable +1 weak facilitator +2 intermediate facilitator +3 strong facilitator

| **Factors** | **-3** | **-2** | **-1** | **0** | **+1** | **+2** | **+3** |
| --- | --- | --- | --- | --- | --- | --- | --- |
| **Consent rate** |  |  |  |  |  |  |  |
| **Familiarity with experimental treatment** |  |  |  |  |  |  |  |
| **Patients’/parents’ attitude towards their taking experimental medicine or placebo** |  |  |  |  |  |  |  |
| **Patients’/parents’ preference for a particular treatment** |  |  |  |  |  |  |  |
| **Patients’/parents’ concerns about side effects of new drug** |  |  |  |  |  |  |  |
| **Duration of trial and follow up** |  |  |  |  |  |  |  |
| **Treatment choice by random allocation** |  |  |  |  |  |  |  |
| **Additional trial investigations** |  |  |  |  |  |  |  |
| **Additional travel and extra costs** |  |  |  |  |  |  |  |
| **Intervention available only in the trial** |  |  |  |  |  |  |  |
| **Communication between research team and patient/parents** |  |  |  |  |  |  |  |
| **Clinician influence** |  |  |  |  |  |  |  |
| **Language or cultural barriers** |  |  |  |  |  |  |  |

**8. Clinical Team factors affecting recruitment**

#### Listed below are *clinical team factors* that commonly affect recruitment. Please indicate whether a listed factor was a facilitator or barrier to recruitment to the trial and rate them from -3 to +3 as below: -3 strong barrier -2 intermediate barrier -1 weak barrier 0 not applicable +1 weak facilitator +2 intermediate facilitator +3 strong facilitator

| **Factors** | **-3** | **-2** | **-1** | **0** | **+1** | **+2** | **+3** |
| --- | --- | --- | --- | --- | --- | --- | --- |
| **Research experience of clinical team** |  |  |  |  |  |  |  |
| **Presence of designated research nurse/practitioner** |  |  |  |  |  |  |  |
| **Availability of designated research team** |  |  |  |  |  |  |  |
| **Availability of research staff out of hours** |  |  |  |  |  |  |  |
| **Shift patterns of work** |  |  |  |  |  |  |  |
| **Motivation of clinical team** |  |  |  |  |  |  |  |
| **Clinical workload** |  |  |  |  |  |  |  |
| **Perceived importance of research generally in clinical practice** |  |  |  |  |  |  |  |
| **Perceived importance of the particular research question** |  |  |  |  |  |  |  |
| **Communication skills of clinical team** |  |  |  |  |  |  |  |
| **Clinician preference for particular treatment** |  |  |  |  |  |  |  |
| **Clinician attitude to involving patients in research** |  |  |  |  |  |  |  |
| **Difficulty in approaching patients for consent** |  |  |  |  |  |  |  |

**9. Information and consent related factors affecting recruitment**

#### Listed below are *information ad consent related factors* that commonly affect recruitment. Please indicate whether a listed factor was a facilitator or barrier to recruitment to the trial and rate them from -3 to +3 as below: -3 strong barrier -2 intermediate barrier -1 weak barrier 0 not applicable +1 weak facilitator +2 intermediate facilitator +3 strong facilitator

| **Factors** | **-3** | **-2** | **-1** | **0** | **+1** | **+2** | **+3** |
| --- | --- | --- | --- | --- | --- | --- | --- |
| **Amount and complexity of trial information provided** |  |  |  |  |  |  |  |
| **Clarity in presentation of trial information** |  |  |  |  |  |  |  |
| **Social and emotional dynamics of trial discussion** |  |  |  |  |  |  |  |
| **Time and setting of consent seeking** |  |  |  |  |  |  |  |
| **Senior doctors and nurses seeking consent** |  |  |  |  |  |  |  |
| **Experience and training of clinical team seeking consent** |  |  |  |  |  |  |  |

**10. Study team factors affecting recruitment**

#### Listed below are *study team factors* that commonly affect recruitment. Please indicate whether a listed factor was a facilitator or barrier to recruitment to the trial and rate them from -3 to +3 as below: -3 strong barrier -2 intermediate barrier -1 weak barrier 0 not applicable +1 weak facilitator +2 intermediate facilitator +3 strong facilitator

| **Factors** | **-3** | **-2** | **-1** | **0** | **+1** | **+2** | **+3** |
| --- | --- | --- | --- | --- | --- | --- | --- |
| **Motivation of the study team at site** |  |  |  |  |  |  |  |
| **Communication and coordination between study team members at site** |  |  |  |  |  |  |  |
| **Communication and coordination between study team at site and CTU** |  |  |  |  |  |  |  |
| **Research experience of PI and study team members at site** |  |  |  |  |  |  |  |

|  |  |  |  |  |  |  |
| --- | --- | --- | --- | --- | --- | --- |
|  |  |  |  |  |  |  |
|  |  |  |  |  |  |  |
|  |  |  |  |  |  |  |
|  |  |  |  |  |  |  |
|  |  |  |  |  |  |  |
|  |  |  |  |  |  |  |
|  |  |  |  |  |  |  |
|  |  |  |  |  |  |  |
|  |  |  |  |  |  |  |
|  |  |  |  |  |  |  |
|  |  |  |  |  |  |  |
|  |  |  |  |  |  |  |
|  |  |  |  |  |  |  |
|  |  |  |  |  |  |  |
|  |  |  |  |  |  |  |
|  |  |  |  |  |  |  |
|  |  |  |  |  |  |  |
|  |  |  |  |  |  |  |
|  |  |  |  |  |  |  |
|  |  |  |  |  |  |  |
|  |  |  |  |  |  |  |
|  |  |  |  |  |  |  |
|  |  |  |  |  |  |  |
|  |  |  |  |  |  |  |
|  |  |  |  |  |  |  |

|  |  |  |  |  |  |
| --- | --- | --- | --- | --- | --- |
|  |  |  |  |  |  |
|  |  |  |  |  |  |
|  |  |  |  |  |  |
|  |  |  |  |  |  |

### 11. What interventions or strategies were applied to overcome any hurdles identified in previous questions and how effective were these?

### 12. How would you organise the trial differently to improve recruitment? Please include additional comments, if any
